# A feasibility pilot study on the effects of a combined cognitive and physical intervention program in older adults in Panama

**DOI:** 10.3389/frdem.2026.1805165

**Published:** 2026-07-20

**Authors:** Elianne Pauli-Quiros, Stephanie Lammie, María Fernanda Martinez, Adam E. Tratner, Sofía Rodríguez-Araña, Giselle A. Rangel, Natalia López P., Alcibiades E. Villarreal, Jemila Juarez, Hjalmar Jones, Gabrielle B. Britton, Diana C. Oviedo-Céspedes

**Affiliations:** 1 Instituto de Investigaciones Científicas y Servicios de Alta Tecnología (INDICASAT-AIP), Panama City, Panama; 2 School of Psychology, Universidad Santa Maria La Antigua (USMA), Panama City, Panama; 3 School of Psychology, Universidad Latina de Panamá, Panama City, Panama; 4 Centro de Vacunación e Investigación (CEVAXIN), Panama City, Panama; 5 Faculty of Psychology, Universidad de Panamá (UP), Panama City, Panama; 6 Sistema Nacional de Investigación (SNI), Panama City, Panama; 7 Florida State University (FSU), Panama City, Panama; 8 Center of Population Sciences for Health Empowerment, Florida State University (FSU), Tallahassee, FL, United States; 9 Clínica Neuropsicológica de Panamá, Panama City, Panama; 10 Caja del Seguro Social (CSS), Panama City, Panama; 11 Centro Psicológico Especializado, Panama City, Panama; 12 Centro Neuropsic, Panama City, Panama

**Keywords:** aging, cognition, cognitive impairment, dementia, non-pharmacological intervention, physical activity, prevention

## Abstract

**Introduction:**

Latin America and the Caribbean are experiencing rapid population aging, accompanied by rising dementia prevalence. Risk is heightened by social inequalities, low education, and limited healthcare resources. While pharmacological treatments for dementia prevention offer only modest benefits, non-pharmacological multimodal interventions have shown promise for maintaining cognition. However, such programs have not yet been evaluated in Panama. This pilot study examined the feasibility and effects of a multimodal physical and cognitive intervention program on cognition, depressive symptoms, and physical performance in community-dwelling older adults in Panama.

**Methods:**

Forty-three sedentary adults aged 60–80 were divided into three groups: combined cognitive–physical intervention group (CG; *n* = 15), a physical intervention group (PG; *n* = 15), or an active control group (AG; *n* = 13) for 4 months. Outcomes included cognition (Montreal Cognitive Assessment, MoCA), depressive symptoms (Geriatric Depression Scale, GDS-15), and physical performance (Short Physical Performance Battery, SPPB), assessed pre- and post-intervention.

**Results:**

Mixed-model ANCOVAs, adjusted for age and education, revealed no significant group-by-time interactions for MoCA, GDS-15, or SPPB. Nonetheless, descriptive analyses indicated the greatest numerical improvements in the CG (MoCA: +3.4 points; GDS-15: −1.1 points). Repeated-measures ANOVAs showed significant overall gains in cognition [*F* (1,40) = 4.733, *p* = 0.036] and small improvements in SPPB [*F* (1,40) = 4.254, *p* = 0.046], though pairwise differences in SPPB were nonsignificant.

**Discussion:**

This study demonstrates the feasibility of delivering multimodal interventions in Panama. Preliminary trends suggest potential cognitive and emotional benefits of combined approaches. Larger, longer trials are needed to confirm efficacy and optimize intervention design.

## 1 Introduction

Population aging is advancing rapidly in low- and middle-income countries, with Latin America and the Caribbean (LAC) experiencing faster demographic change than other world regions (Naciones Unidas, 2022). As a result, the prevalence of neurodegenerative conditions such as Alzheimer’s disease (AD), the leading cause of dementia, continues to rise (Alzheimer’s Association, 2024a). In LAC, this burden is further intensified by a high prevalence of cardiovascular risk factors, low educational attainment, persistent social inequalities, and limited access to healthcare services, while available pharmacological treatments offer only modest benefits for symptom reduction or disease modification (Parra et al., 2021; Takramah and Asem, 2022).

Evidence suggests that up to 56% of dementia cases in the region may be attributable to modifiable risk factors such as low childhood education, depression, alcohol use, social isolation, and physical inactivity (Livingston et al., 2024). Among these, physical inactivity is one of the most prevalent and actionable risk factors, particularly in older adults residing in urban settings in LAC (Guthold et al., 2018). A growing body of evidence indicates that regular physical activity is associated with better cognitive performance, reduced risk of cognitive decline, and delayed onset of dementia through mechanisms involving improved cerebral blood flow, neurogenesis, synaptic plasticity, and reductions in inflammation and cardiometabolic risk (Ahlskog et al., 2011; Erickson et al., 2011; Northey et al., 2018; World Health Organization, 2020).

Randomized controlled trials and meta-analyses have demonstrated that aerobic exercise, resistance training, balance, and multicomponent physical programs can improve executive function, processing speed, memory, mobility, and overall physical functioning in cognitively healthy older adults and those with MCI (Blondell et al., 2014; Sanders et al., 2019). Importantly, combined aerobic and strength-based interventions appear to yield the most consistent cognitive benefits, particularly when delivered at moderate intensity and sustained over several months (Ahlskog et al., 2011). In addition to cognitive outcomes, physical interventions have been shown to improve mood, reduce depressive symptoms, enhance sleep quality, and lower fall risk, all of which are closely linked to cognitive and functional trajectories in aging populations (Ungvari et al., 2023; Castellote-Caballero et al., 2024).

Evidence-based preventive strategies, such as physical activity, healthy diets, cognitive stimulation, and management of cardiovascular risk, show substantial potential to delay cognitive decline and promote mental well-being in older adults (World Health Organization, 2020). Non-pharmacological interventions (NPIs), which prioritize safety, accessibility, and low risk, aim to slow cognitive deterioration, reduce neuropsychiatric symptoms, and lessen caregiver burden (Poulos et al., 2017). Multimodal NPIs that integrate physical exercise with cognitive training and lifestyle modification appear to have synergistic effects, addressing multiple dementia risk pathways simultaneously (Zucchella et al., 2018).

Multidomain interventions combining physical, cognitive, nutritional, and vascular risk management components have demonstrated consistent cognitive benefits, with the Finnish Geriatric Intervention Study to Prevent Cognitive Impairment and Disability (FINGER) providing landmark evidence of slowed cognitive decline in at-risk older adults (Ngandu et al., 2015). In LAC, smaller randomized and quasi-experimental studies have also reported cognitive, emotional, and physical health improvements following multidomain interventions that include structured physical activity programs (Coelho et al., 2013; López et al., 2015). Building on this evidence, the LATAM-FINGERS trial is currently evaluating the feasibility and effectiveness of culturally adapted multidomain interventions across 14 countries in the region (Alzheimer’s Association, 2024b). However, to date, no such multimodal intervention has been empirically examined in Panama, despite projections of a 449% increase in dementia cases by 2050 (Nichols et al., 2022).

Although Panama is classified as a middle- to high-income country, it continues to exhibit some of the highest levels of income inequality worldwide (López Marmolejo et al., 2023; World Bank Organization, 2026). Compared to other Central American countries, Panama is projected to experience a greater increase in dementia prevalence, a trend attributed to socioeconomic inequality and health disparities (Nichols et al., 2022). Many Panamanians report skipping medical appointments or prescribed treatments due to the high costs of private healthcare (Leon, 2003; Macinko et al., 2016; Greene and Guanais, 2018) as well as barriers to accessing healthcare in rural areas (Romero and Quental, 2014; Baird, 2023). In addition, Panama’s public healthcare system is strained by limited funding, poor infrastructure, and frequent shortages of medication and staff. Together, these factors contribute to the high prevalence of chronic conditions, such as hypertension, diabetes, and obesity, as many individuals lack timely access to effective care (Del Rio et al., 2022; Quintana et al., 2023). Panama also has an underdeveloped research ecosystem, resulting in lower investment in scientific research and a diminished interest from the general population to participate in clinical studies, both of which hinder the development of aging research and the implementation of effective preventive strategies (Romero and Quental, 2014; Crespo, 2025). Older adults in Panama are still underrepresented in research on aging and face multiple, overlapping vulnerabilities, and age-related discrimination [Instituto Nacional de Estadística y Censo (INEC), 2015]. In addition, limited opportunities for retirees to engage in recreational, cognitive, and physical activities may further contribute to an increased risk of cognitive decline (Camargo, 2015; Villar Liste, 2025).

As prevention research targeting aging is limited in Panama, feasibility pilot studies are fundamental to determine whether a larger definitive study can be successfully implemented, and they typically evaluate multiple methodological, logistical, and practical factors (Leon et al., 2011). Common aspects assessed include the ability to recruit and retain participants, adherence to the intervention protocol, acceptability of the procedures to participants and staff, adequacy of measurement tools, time required for data collection, availability of resources, and potential safety concerns (Thabane et al., 2010). Pilot studies may also examine barriers related to funding, personnel, infrastructure, and participant burden. Together, these factors help researchers refine the study design, optimize procedures, and reduce the risk of failure in subsequent full-scale trials (Eldridge et al., 2016; Pearson et al., 2020).

This pilot study aimed to evaluate the effects of a multimodal intervention program on cognitive functioning, subjective well-being, and physical health in community-dwelling older adults in Panama, and to determine the feasibility of implementing such a program in this context. We hypothesized that participants receiving the combined cognitive and physical intervention would show greater improvements in cognitive functioning, subjective well-being, and physical health compared to controls, and that outcomes would be superior in the combined intervention group compared to the physical intervention-only and control groups.

## 2 Methods

### 2.1 Participants

**Figure 1:**
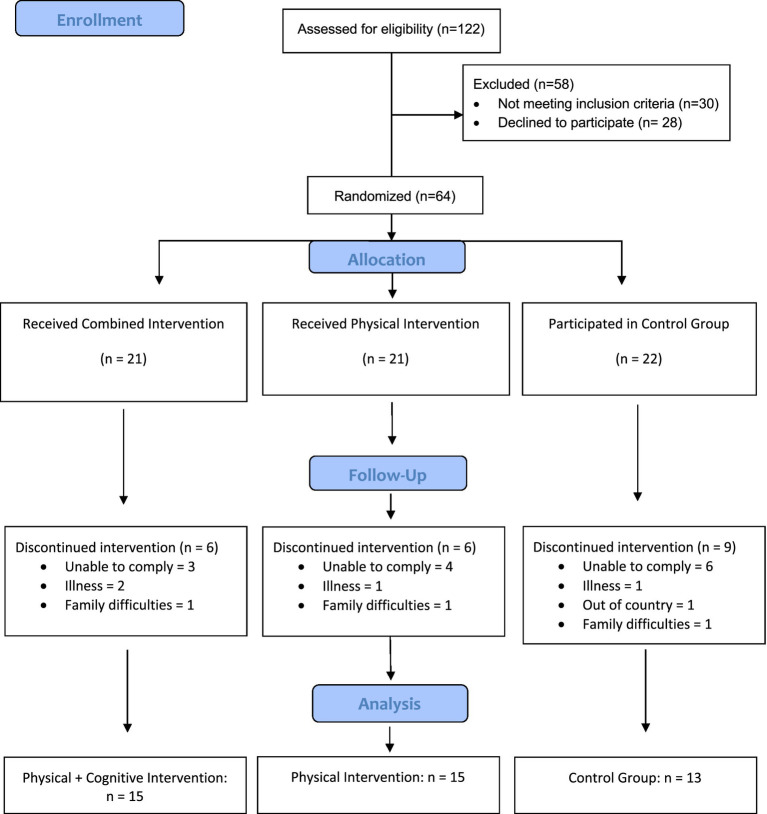
Flowchart of the study’s sample of older adults.

Participants were recruited through active and passive strategies. Passive strategies included social media flyers and posts, announcements at science outreach events, and partnerships with churches, retiree groups and senior community gatherings. Active strategies included outreach to potential participants. Individuals were contacted through referral or because they were included in our participant databases to determine whether they met inclusion criteria and if they were interested. Inclusion criteria were being a resident of Panama City, aged 60–80 years, sedentary, and scoring ≥20 on the Montreal Cognitive Assessment (MoCA). We performed a power analysis using G*Power (Faul et al., 2007), which recommended a minimum of 66 participants for mixed model ANOVA and 54 participants for the repeated measures ANOVA used. After initially recruiting 64 participants for the study, 21 participants were lost to attrition, resulting in a final sample size of 43 participants. The most common reason for withdrawal was inability to comply with the study activities (*n* = 13), followed by illness (*n* = 4), family-related difficulties (*n* = 3), and travel outside the country (*n* = 1). Participant flow and reasons for withdrawal are presented in Figure 1. All participants provided written informed consent, and the study was approved by the Research Bioethics Committee of Universidad Santa María La Antigua (CBI-USMA), code 2022-P002.

### 2.2 Materials and procedure

At baseline, participants completed a sociodemographic interview. Cognitive functioning was assessed in person using the MoCA, and depressive symptoms were measured with the Geriatric Depression Scale (GDS-15). Physical measures included height, weight, blood pressure, abdominal circumference, and grip strength (via dynamometer). Functional mobility was evaluated with the Short Physical Performance Battery (SPPB), which includes measures of balance, gait speed, and lower limb strength. All assessments were conducted at baseline and repeated at a four-month follow-up following completion of the intervention.

Participants were assigned to one of three groups for a four-month intervention period. The combined intervention group (CG; *n* = 15) received both physical and cognitive training. The cognitive component included computerized cognitive training using the CogniFit platform, which participants completed individually three to four times per week in sessions lasting approximately 12–20 min. Prior to initiating the program, participants attended an orientation session at the research center where they received instruction on how to use the platform, activate their accounts, and perform the training exercises. Technical support was available throughout the intervention via phone or text message. In addition to the computerized training, participants attended weekly in-person group cognitive sessions lasting approximately 2 h each (approximately 8 h per month). These sessions were facilitated by trained research staff and included structured activities targeting multiple cognitive domains, including memory, attention, language, gnosis, praxis, and executive functioning. The activities were delivered using a variety of formats, including paper-and-pencil tasks, audiovisual materials, interactive exercises, and board games designed to promote cognitive engagement and group interaction.

The physical training component consisted of a structured moderate-intensity aerobic exercise program based on brisk walking, performed independently three to five times per week for approximately 30 min per session. This corresponded to approximately 1.5–2.5 h of walking per week. The program was supplemented with monthly group walks supervised by the research team. Physical activity was monitored using a smartwatch and mobile application that recorded step count, distance, calories expended, and heart rate. Participants were asked to record these metrics in a written log, which was later compared with the data registered in the mobile application to monitor adherence. The physical intervention group (PG; *n* = 15) followed the same physical training protocol described above but did not receive the cognitive training component. The active control group (AG; *n* = 13) attended monthly educational sessions led by invited experts on topics related to aging and health, including healthy nutrition, sleep quality, social determinants of health, and the management of cardiovascular risk factors. The intervention protocol was standardized and maintained consistently throughout the four-month intervention period, without modifications to frequency or duration.

Feasibility-related outcomes were explored through post-intervention satisfaction scales. Satisfaction was assessed at the end of the intervention using structured questionnaires administered to participants in all study groups (combined intervention, physical intervention, and control group) after each of the group sessions. The questionnaires evaluated overall satisfaction with the program, perceived adequacy of the intervention duration and session frequency, clarity and quality of the content, perceived usefulness of the information provided, and satisfaction with specific components of the intervention, including group activities, digital tools (smartwatch use), and computerized cognitive training. Participants were also asked whether the program met their expectations and whether they would recommend it to others.

## 3 Results

### 3.1 Baseline characteristics

Table 1 summarizes the demographic characteristics of the final sample (*n* = 43). One-way ANOVA analyses revealed no significant differences among the combined intervention (CG), physical intervention (PG) and active control (AG) groups across demographic, physical, or clinical variables, indicating comparability for subsequent analyses. Additionally, one-way ANOVAs were used to evaluate group differences in cognitive function (MoCA), depressive symptoms (GDS-15), and physical performance (SPPB) at baseline. Results indicated no significant group differences in MoCA [*F* (2, 42) = 1.489, MSE = 8.754, *p* = 0.238], GDS-15 [*F* (2, 42) = 0.998, MSE = 5.806, *p* = 0.378], or SPPB [*F* (2, 42) = 0.114, MSE = 0.179, *p* = 0.893] scores at baseline.

**Table 1:** Demographic characteristics of participants according to intervention group (final sample).

Variable category	Total (*n* = 43)	Combined (*n* = 15)	Physical (*n* = 15)	Control (*n* = 13)	*p*
	*n* (%)/M (SD)	*n* (%)/M (SD)	*n* (%)/M (SD)	*n* (%)/M (SD)	
Age	68.2 (4.9)	70.2 (5.2)	66.5 (4.7)	67.9 (4.4)	0.114
Sex					
Women	33 (76.7%)	12 (80.0%)	13 (86.7%)	8 (61.5%)	0.272
Men	10 (23.3%)	3 (20.0%)	2 (13.3%)	5 (38.5%)	
Years of education	18.2 (3.4)	18.6 (2.3)	19.0 (3.3)	16.7 (4.2)	0.154
Monthly family income (USD)					
<500	1 (2.3%)	1 (6.7%)	0 (0.0%)	0 (0.0%)	0.650
501–1,200	12 (27.9%)	5 (33.3%)	4 (26.7%)	3 (23.1%)	
>1,200	30 (69.8%)	9 (60.0%)	11 (73.3%)	10 (76.9%)	
Marital status					
No partner	20 (46.5%)	7 (46.7%)	8 (53.3%)	5 (38.5%)	0.734
With partner	23 (53.5%)	8 (53.3%)	7 (46.7%)	8 (61.5%)	
Residence					
Lives alone	5 (11.6%)	2 (13.3%)	2 (13.3%)	1 (7.7%)	0.990
With family	32 (74.4%)	11 (73.3%)	11 (73.3%)	10 (76.9%)	
In company	6 (14.0%)	2 (13.3%)	2 (13.3%)	2 (15.4%)	
Chronic diseases					
Diabetes	7 (16.3%)	4 (26.7%)	2 (13.3%)	1 (7.7%)	0.370
Hypertension	25 (58.1%)	8 (53.3%)	10 (66.7%)	7 (53.8%)	0.709
Asthma	3 (7.0%)	0 (0.0%)	2 (13.3%)	1 (7.7%)	0.355
Cancer	3 (7.0%)	2 (13.3%)	1 (6.7%)	0 (0.0%)	0.385
Cardiovascular	4 (9.3%)	1(6.7%)	2 (13.3%)	1 (7.7%)	0.798
Chronic obstructive pulmonary disease	5 (11.6%)	2 (13.3%)	0 (0.0%)	3 (23.1%)	0.159
Chronic kidney disease	3 (7.0%)	0 (0.0%)	1 (6.7%)	2 (15.4%)	0.280
Arthritis	5 (11.6%)	1 (6.7%)	3 (20.0%)	1 (7.7%)	0.454
Osteoporosis	12 (27.9%)	5 (33.3%)	3 (20.0%)	4 (30.8%)	0.691
Smoke/smoked					
Yes	21 (48.8%)	9 (60.0%)	6 (40.0%)	6 (46.2%)	0.534
Sleep problems					
Yes	19 (44.2%)	6 (40.0%)	9 (60.0%)	4 (30.8%)	0.276
Subjective cognitive impairment					
Yes	24 (55.8%)	7 (46.7%)	11 (73.3%)	6 (46.2%)	0.238
Number of medications					
No polypharmacy (<4)	22 (51.2%)	8 (53.3%)	9 (60.0%)	5 (38.5%)	0.661
Polypharmacy (5–7)	15 (34.9%)	4 (26.7%)	5 (33.3%)	6 (46.2%)	
Excess polypharmacy (>8)	6 (14.0%)	3 (20.0%)	1 (6.7%)	2 (15.4%)	
ApoE4 carrier					
Yes	5 (11.6%)	3 (20.0%)	1 (6.7%)	1 (7.7%)	0.454

### 3.2 Mixed model analyses

Mixed-model analyses of covariance (ANCOVAs), controlling for age and education, examined the effect of the intervention on cognitive function (MoCA), depressive symptoms (GDS-15), and physical performance (SPPB) (Table 2). No significant group and time interactions were observed for any outcome measure: MoCA [*F* (2, 38) = 1.851, MSE = 3.694, *ηp*^2^ = 0.089, *p* = 0.171], GDS-15 [*F* (2, 38) = 0.012, MSE = 0.033, *ηp*^2^ = 0.001, *p* = 0.988], or SPPB [*F* (2, 38) = 0.035, MSE = 0.045, *ηp*^2^ = 0.002, *p* = 0.965]. (*F* = 0.035, *p* = 0.965). Despite the absence of statistically significant interaction effects, the descriptive examination of groups means revealed patterns consistent with the study’s hypotheses. The combined intervention group demonstrated the largest numerical improvement in cognitive function, with MoCA scores increasing from 22.9 (SD = 2.7) at baseline to 26.3 (SD = 2.0) post intervention. The physical intervention group showed a modest increase in MoCA scores (M = 25.3, SD = 2.9 to M = 25.9, SD = 3.1), while the control group also improved slightly (M = 24.1, SD = 1.3 to M = 25.2, SD = 2.2). For depressive symptoms, the combined intervention group also demonstrated the greatest reduction, with GDS-15 scores decreasing from 2.5 (SD = 2.2) at baseline to 1.4 (SD = 1.9). Smaller decreases were observed in the physical intervention group (M = 1.7, SD = 1.7 to M = 1.2, SD = 1.7), while the control group improved from 3.0 (SD = 3.2) to 2.0 (SD = 2.0). For physical performance (SPPB), all groups demonstrated minimal change. The combined group maintained a mean score of 9.9 from Time 1 (SD = 1.2) to Time 2 (SD = 1.5), the physical group improved slightly from 9.7 (SD = 1.2) to 10.0 (SD = 1.6), and the control group showed a modest improvement from 9.9 (SD = 1.2) to 10.3 (SD = 1.4).

**Table 2:** Mixed model analyses of variance.

Test	Group	Time 1	Time 2	F	*p*
		M (SD)	M (SD)		
MoCA	Combined	22.9 (2.7)	26.3 (2.0)	1.851	0.171
	Physical	25.3 (2.9)	25.9 (3.1)		
	Control	24.1 (1.3)	25.2 (2.2)		
GDS-15	Combined	2.5 (2.2)	1.4 (1.9)	0.012	0.988
	Physical	1.7 (1.7)	1.2 (1.7)		
	Control	3.0 (3.2)	2.0 (2.0)		
	Combined	9.9 (1.2)	9.9 (1.5)	0.035	0.965
SPPB	Physical	9.7 (1.2)	10.0 (1.6)		
	Control	9.9 (1.2)	10.3 (1.4)		

### 3.3 Time effects across groups

To examine overall changes irrespective of intervention group, repeated-measures ANOVAs were conducted (Table 3). The first model comparing MoCA scores was statistically significant [*F* (1, 40) = 4.733, MSE = 9.847, *ηp*^2^ = 0.106, *p* = 0.036], and pairwise comparisons showed that MoCA scores increased significantly from baseline (M = 24.47, SD = 2.453) to follow-up (M = 25.84, SD = 2.468) (*p* < 0.001). The second model comparing GDS-15 scores between time points [*F* (1, 40) = 1.157, MSE = 2.967 *ηp*^2^ = 0.028, *p* = 0.289] was not statistically significant, however GDS-15 scores decreased slightly from baseline (M = 2.40; SD = 2.41) to follow-up (M = 1.51; SD = 1.86). The third model comparing SPPB between time points was statistically significant [*F* (1, 40) = 4.254, MSE = 5.172, *ηp*^2^ = 0.096, *p* = 0.046], however, SPPB scores only increased slightly and pairwise comparisons indicated that the means did not differ significantly from time 1 (M = 9.79, SD = 1.23) to follow-up (M = 10.09, SD = 1.49) (*p* = 0.211).

**Table 3:** Repeated measures analyses of variance.

Test	Time 1	Time 2	*F*	*p*
	M (SD)	M (SD)		
MoCA	24.47 (2.45)	25.84 (2.46)	4.733	0.036
GDS-15	2.40 (2.41)	1.51 (1.86)	1.157	0.289
SPPB	9.79 (1.23)	10.09 (1.49)	4.254	0.046

## 4 Discussion

This pilot study evaluated the preliminary cognitive, emotional, and physical effects of cognitive, physical, and combined interventions and, importantly, demonstrated that a multimodal program is feasible to implement in community-dwelling older adults in Panama. Although group-by-time interactions were not statistically significant, the combined intervention group showed the largest numerical gains in cognition, consistent with evidence that both computerized and paper-based cognitive training can strengthen global cognition (Santos et al., 2015; Vale et al., 2018). Exercise supports respiratory, cognitive, cardiovascular, and musculoskeletal health, whereas cognitive training promotes neuroplasticity, which jointly enhance the efficacy of multimodal intervention designs (Zucchella et al., 2018).

Across all participants, global cognition improved significantly over time, reinforcing prior research showing that structured, socially engaging activities can yield meaningful cognitive enrichment (Haslam et al., 2014; Train the Brain Consortium et al., 2017). Depressive symptoms also declined from baseline to follow-up, though not significantly, aligning with findings that link cognitive and social engagement to improved emotional well-being in older adults (Zülke et al., 2024; Srisuwan et al., 2025). In contrast, physical performance showed minimal gains, likely due to the exclusive focus on aerobic training, whereas prior multimodal regimens that also include anaerobic training demonstrated broader functional improvements (Booth et al., 2016; Laatar et al., 2018; Langoni et al., 2019), suggesting that future iterations of this program could benefit from a more diversified physical training component. Previous research indicates that physical intervention programs targeting key domains of health in older adults, such as mobility, balance, muscular endurance, and aerobic conditioning, should incorporate muscle strengthening and balance exercises, either alone or in combination with aerobic training (Sherrington et al., 2008; Da Silveira et al., 2018; Laatar et al., 2018). Moreover, mixed exercise programs have been shown to enhance lower-limb strength, a critical factor in fall prevention among older populations (Gillespie et al., 2012; Pedroso et al., 2012; Booth et al., 2016). Consistent with prior multimodal regimens demonstrating broader functional improvements (Laatar et al., 2018; Langoni et al., 2019), future iterations of this program may benefit from a more comprehensive and diversified physical training component to optimize functional outcomes.

This study also provides preliminary insight into participants’ experience with the program through post-intervention satisfaction. High levels of satisfaction were observed across all intervention groups, with most participants reporting being “very satisfied” with the program. Core aspects of the intervention, including duration, session frequency, and content quality, were rated positively. Participants also reported favorable experiences with specific components, such as group-based activities, smartwatch-guided physical activity, and computerized cognitive training via CogniFit. Furthermore, all participants indicated that the program met their expectations and that they would recommend it to others, suggesting perceived usefulness and positive effects on their daily routines. Future studies should incorporate systematic evaluation of complimentary factors such as adherence and barriers to participation to better asses the feasibility of multimodal interventions in community-dwelling older adults.

In addition to participant satisfaction, several methodological indicators support the feasibility of conducting this type of multimodal intervention with community-dwelling older adults in Panama. Recruitment strategies combining passive dissemination and active outreach proved effective for enrolling participants within the targeted age range, although the study also revealed important challenges related to retention. Despite reaching the minimum sample size estimated in the power analysis at baseline, attrition reduced the final sample, with the most frequent reasons for withdrawal being difficulty adhering to the intervention schedule, health problems, family responsibilities, and travel. These findings highlight common barriers in behavioral and lifestyle interventions with older adults and suggest the need for different strategies to improve attrition rates. Future studies could incorporate more and better incentives that could range from flexible schedules and strategies to reduce participant burden in future trials, to transportation support and rewards such as gift cards (considering ethical considerations). Workshops and other educational materials that can benefit participants can also be included to increase trust and engagement with participants.

Participants demonstrated adherence to the intervention protocol, and the procedures and schedules involved in the project were well accepted. The implementation of computerized cognitive training, group cognitive sessions, and independently performed aerobic exercise monitored through wearable devices was completed without major difficulties, indicating that the required infrastructure, personnel, technological resources, and data collection procedures were appropriate and feasible within this setting. In feasibility and pilot studies, aspects such as recruitment capacity, retention rates, adherence to the protocol, acceptability of procedures, adequacy of outcome measures, and logistical demands are critical for determining whether a full-scale trial is viable (Thabane et al., 2010; Leon et al., 2011; Eldridge et al., 2016). Taken together, the present findings suggest that a larger randomized controlled trial is feasible in the Panamanian context, although future studies should incorporate strategies to minimize attrition and optimize adherence to ensure sufficient statistical power.

Several limitations should be considered when interpreting these preliminary findings. Recruitment and retention difficulties resulted in a relatively small sample size, which limited statistical power and reduced the ability to detect significant effects, particularly in the mixed-model analyses. The small sample limited the data available to evaluate feasibility outcomes as well as the generalizability findings, as the study participants were not representative of the broader population of older adults in low- and middle-income countries across LAC. In particular, the sample was mostly female and reported relatively high levels of education and income, thereby limiting the applicability of the findings across sociodemographic groups. Different strategies to ensure a more heterogeneous sample could include an active promotion of accessibility and identification of recruitment barriers. Collaborations with organizations and community leaders of underserved populations and compensation mechanisms for participants that cannot access the research facilities are necessary to improve recruitment representativeness. Although our research team is diverse, it is imperative to include project collaborators that have access to different demographic groups from a range of socioeconomic backgrounds. Another identified limitation was that baseline levels of depressive symptoms were generally low, and physical functioning was relatively preserved, potentially constraining the magnitude of measurable change in these domains. Furthermore, improvements observed in cognition and well-being may have been partially influenced by extraneous factors, including increased social interaction, repeated exposure to assessments, or the novelty of participating in structured activities. Nevertheless, despite recruitment difficulties, adherence rates were high, with participants demonstrating strong engagement and reporting perceived benefits from the intervention.

## 5 Conclusion

Despite limitations, the feasibility outcomes emerge as an encouraging aspect of this study. High rates of enrollment, retention, and participation demonstrate that older adults in Panama can successfully engage with a structured program integrating cognitive training, digital health tools, walking routines, and group-based activities. These findings indicate that community-based multimodal interventions are not only acceptable but also operationally viable in this context, a critical step for expanding mental health and dementia-prevention strategies in low- and middle-income countries. Results provide critical groundwork for future studies with large and more diverse samples, the inclusion of additional intervention components, increased intervention intensity, and longer follow-up periods. As the first multimodal intervention study for the prevention of cognitive decline conducted in Panama, this work contributes to the growing body of aging and mental health research in Latin America and represents an important step toward developing scalable strategies to support cognitive health in the region.

## Data Availability

The raw data supporting the conclusions of this article will be made available by the authors, without undue reservation.
